# Effect of neoadjuvant chemotherapy on the immune microenvironment in gastric cancer as determined by multiplex immunofluorescence and T cell receptor repertoire analysis

**DOI:** 10.1136/jitc-2021-003984

**Published:** 2022-03-31

**Authors:** Xiaofang Xing, Jinyao Shi, Yongning Jia, Yunsheng Dou, Zhongwu Li, Bin Dong, Ting Guo, Xiaojing Cheng, Xiaomei Li, Hong Du, Ying Hu, Shuqin Jia, Jian Zhang, Ziyu Li, Jiafu Ji

**Affiliations:** 1 Department of Gastrointestinal Translational Research, Key Laboratory of Carcinogenesis and Translational Research (Ministry of Education), Peking University Cancer Hospital, Beijing, China; 2 Department of Gastrointestinal Surgery, Key Laboratory of Carcinogenesis and Translational Research (Ministry of Education), Peking University Cancer Hospital & Institute, Beijing, China; 3 Academy of Medical Engineering and Translational Medicine, Tianjin University, Tianjin, China; 4 Department of Pathology, Key Laboratory of Carcinogenesis and Translational Research (Ministry of Education), Peking University Cancer Hospital & Institute, Beijing, China; 5 Biobank, Key Laboratory of Carcinogenesis and Translational Research (Ministry of Education), Peking University Cancer Hospital & Institute, Beijing, China; 6 Department of Molecular Diagnosis, Key Laboratory of Carcinogenesis and Translational Research (Ministry of Education), Peking University Cancer Hospital & Institute, Beijing, China

**Keywords:** gastrointestinal neoplasms, tumor microenvironment, tumor biomarkers

## Abstract

**Background:**

The combination of immune checkpoint blockade and chemotherapy has revolutionized the treatment of advanced gastric cancer (GC). It is crucial to unravel chemotherapy-induced tumor microenvironment (TME) modulation and identify which immunotherapy would improve antitumor effect.

**Methods:**

In this study, tumor-associated immune cells (TAICs) infiltration in residual tumor after neoadjuvant chemotherapy (NAC) together with 1075 cases of treatment-naïve GC patients was analyzed first. Then we performed multiplex fluorescence staining of a panel of immune markers (CD3, CD4, CD8, FOXP3 and PDL1) and T cell receptor β-chain sequencing to phenotype and enumerate T cell subpopulations and clonal expansion in paired GC samples (prechemotherapy and postchemotherapy) from another cohort of 30 cases of stage II/III GC patients.

**Results:**

Infiltration of CD68^+^ macrophages in residual tumors after NAC was significantly decreased compared with treatment-naïve GC patients, while no significant difference observed with respect to other immune markers. In residual tumors, post-NAC CD8 +T cells and CD68+ macrophages levels were significantly associated with chemotherapy response. Post-NAC CD8+ T cell levels remained as an independent predictor for favorable prognosis. Furthermore, when comparing the paired samples before and after NAC from 30 cases of stage II/III GC patients, we found FOXP3+ regulatory T cells proportion significantly decreased after chemotherapy. Pre-NAC FOXP3+ T reg cells level was much richer in the response group and decreased more significantly in the stromal compartment. CD8+ cytotoxic T lymphocytes levels were elevated after chemotherapy, which was more significant in the group treated with XELOX regimen and in patients with better response, consistent with the TCR diversity elevation.

**Conclusions:**

These findings have deepened our understanding of the immune modulating effect of chemotherapy and suggest that the immune profile of specimens after standard chemotherapy should be considered for the personalized immunotherapy to ultimately improve clinical outcome in patients with GC.

## Background

There are more than one million gastric cancer (GC) cases and an estimated 783 000 GC-related deaths in 2018, making it the fifth most common and the third most deadly cancer globally. Of concern, nearly half of new cases and deaths occur in China.^1 2^ With the rise of immunotherapy, it is expected to change the clinical practice of GC. Recently, Food and Drug Administration (FDA)-approved nivolumab in combination with chemotherapy for the initial treatment of patients with advanced or metastatic GC.^3 4^ It is the first regimen approved combining immunotherapy and chemotherapy for advanced GC. Median survival was 13.8 months for patients who received nivolumab plus chemotherapy compared with 11.6 months for patients who received chemotherapy alone.^5^ However, the mechanisms by which chemotherapy augments the effect of immune checkpoint blockade remain in GC remain elusive. The contribution of various TAICs subtypes to treatment response remains unknown. To date, few studies examined immunological changes in the GC microenvironment after chemotherapy.

Accumulating evidence suggests that cytotoxic agents can modulate the tumor microenvironment (TME) through various mechanisms, such as immunogenic cell death, stimulation of T cell responses and inhibition of tumor-induced immune suppression.^6 7^ Chemotherapy has a potential to trigger immune activation by inducing tumor-associated neoantigen release, which in turn activates antigen-presenting cells such as tumor-associated macrophages (TAMs) and dendritic cells by Toll-like receptors.^8^ Platinum agents such as cisplatin have been reported to increase dendritic cells while depleting myeloid-derived suppressor cells in melanoma mouse models.^9^ It is also reported that NAC was associated with increased infiltration of cytotoxic CD8+ T cells and CD20+ B cells.^10^ Preclinical data revealed that mice vaccinated with cisplatin-treated ovarian cancer cells had enhanced antitumor immunity and improved survival that were largely dependent on CD4+ T cell mediated immune response.^11^ Actually, the interaction between immune cells and tumor cells is more likely based on equilibrium between immune activation and tolerance so that investigation of the immune activating and suppressing cells may be of more informative.

Various aspects of immune cells, such as type, functional polarization and local distribution have been shown to influence clinical outcome in multiple cancer types.^12^ Previous studies reported that high proportion of tumor-infiltrating lymphocytes (TILs), in particular that with cytotoxic function such as CD8+ and natural killer cells are correlated with favorable prognosis in various cancer.^13–16^ On the other hand, immune-suppressive cells, such as FOXP3+ regulatory T cells, are often associated with a negative prognostic impact.^13 17 18^ TAMs have been reported to predict an unfavorable outcome, especially the M2-polarized (CD163+) subset.^14 19^ However, most studies have focused on treatment-naïve GC patients.^16^ Recently, tumor-associated immune cells (TAICs) infiltrations playing a vital role in the response to neoadjuvant chemotherapy (NAC) has been proposed in breast cancer, non-small cell lung cancer (NSCLC), etc,^10 20–23^ but the immune cell subpopulation and its prognostic contributing to chemotherapy response have not been clarified in GC yet.

NAC is increasingly prescribed for patients with locally advanced GC for potentially down-staging the tumor stages and improves survival, and it provides opportunities for monitoring the treatment sensitivity of GC tumors ‘in vivo’. The neoadjuvant setting where paired samples before and after chemotherapy offers a useful model to study the impact of cytotoxic treatment on antitumor immunity.

In this study, TAICs analysis in residual tumor after systemic NAC was performed in a cohort of 314 cases of patients with GC first. Then the multiplex fluorescence staining of a panel of immune markers (CD3, CD4, CD8, FOXP3 and PDL1) and T cell receptor sequence were done in paired GC samples (prechemotherapy and postchemotherapy) from 30 stage II/III GC patients whose response to NAC were rigorously defined.

Our study objectives were as follows: (1) to identify and quantify the impact of NAC on the TME, including subgroups of TAICs and TCR diversity; (2) to figure out the predictive and prognostic values of TAICs levels, subtypes and locations; and (3) to discover possible mechanisms by which chemotherapy may enhance immune response.

## Methods

### Study population

The first study population is consisted of 1416 GC patients who underwent gastrectomy for adenocarcinoma at the stomach or esophagus–stomach junction at Peking University Cancer Hospital. All the samples were independently inspected by two pathologists to confirm the identified pTNM stages. The inclusion criteria were primary diagnosis of GC with available Formalin fixation and paraffin embedding (FFPE) tissues and follow-up information. This study population was divided into two cohorts, 1075 cases without preoperative treatment (cohort 1) and 341 cases treated with NAC (cohort 2). Another independent cohort (cohort 3) is 30 stage II/III GC patients treated with NAC whose response to were rigorously defined. Biopsy tumor tissues prior to NAC and postoperative tissues from patients were obtained in this cohort. The workflow of the study was shown in online supplemental figure S1. NAC cohort received the fluorouracil-based treatment regimen of capecitabine/S-1+oxaliplatin (XELOX (oxaliplatin, 130 mg/m^2^, intravenously, day 1; and capecitabine, 1000 mg/m^2^, orally, days 1–14) or SOX (oxaliplatin 130 mg/m^2^, intravenously, day 1; and S-1, 40–60 mg, twice a day, orally, days 1–14)) for two to four cycles. The pathological features were evaluated according to Mandard TRG score, patients with TRG 0 or 1 who achieved complete or partial response were defined as responders; patients with TRG 2 or 3 were defined as non-responders. The overall survival time was defined to be the period of time in months from the date of surgery to the date of death from any cause. The pTNM stage was determined according to the eighth edition of the UICC-AJCC guidelines.

10.1136/jitc-2021-003984.supp1 Supplementary data

**Figure 1:**
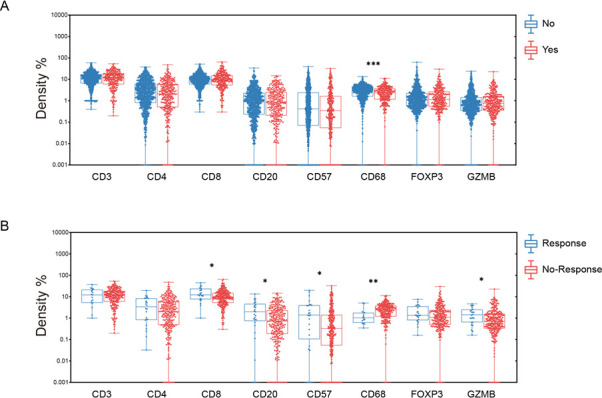
(A) Comparison of immune cell infiltration of gastric cancer in patients treated with and without NAC. (B) Comparison of immune cells infiltration of gastric cancer in patients with different response (TRG 0–1 vs TRG 2–3) to NAC. X-axis represents the immune markers, and Y-axis represents the staining density of each marker. NAC, neoadjuvant chemotherapy.

### Tissue microarray

The TMA used for this study includes 1416 unselected, primary, sporadic GCs. All H&E slides were centrally reviewed to confirm the tumor type and differentiation grade at Department of Pathology in Peking University Cancer Hospital. Representative areas of each tissue sample were identified and carefully marked on H&E-stained sections. Three representative core-tissue specimens (1 mm in diameter) were punched from the corresponding individual donor tissue blocks and rearranged in recipient blocks.

### TAIC analysis

Primary antibodies used in immunostaining were validated and listed in online supplemental figures S2–S4 and table S1. TAICs density was analyzed by automated interpretation. Slides were scanned at ×20 magnification using an Aperio XT digital slide scanner and subjected to automated image analysis to detect and quantify immunoreactivity. TMAi, a software developed in-house, was used to discriminate brown (immunopositive) pixels, blue (immunonegative) pixels, and white (empty space) pixels. All the cores were reviewed after the image analysis by a senior Gastrointestinal (GI) histopathologist to confirm that the brown DAB staining was detected accurately by the software. To exclude the cores without tumor cells, the slides were stained with CK antibody to confirm spots including ≥50% malignant epithelial cells. Percentage immunoreactivity (positive cells/(positive cells+negative cells) *100) from all the available cores per case was averaged and used as a surrogate for the extent of immune cell infiltration. Details of the protocols and scoring schema were as previously described.^16 24^

**Figure 2:**
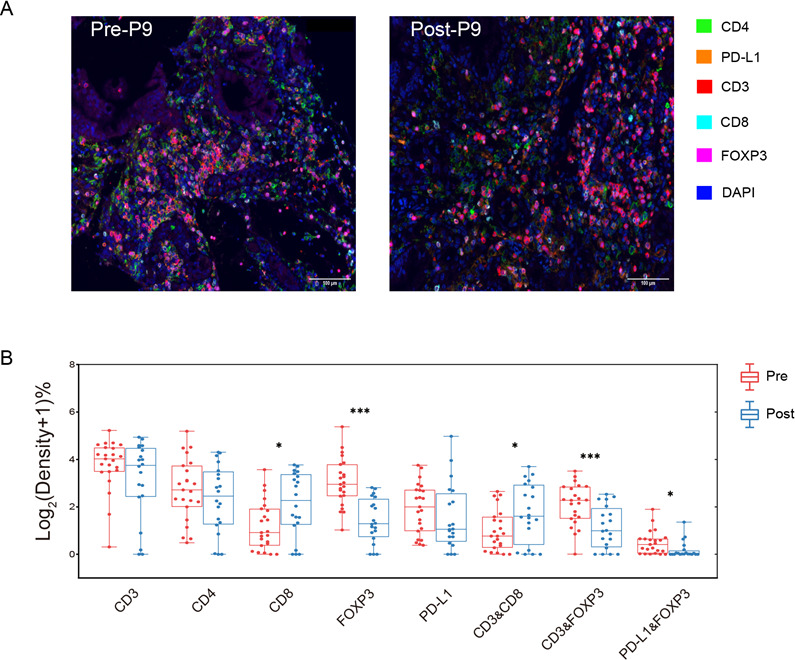
(A) Multiplex immunofluorescence images of immune cell infiltration in paired pre-NAC and post-NAC specimens of gastric cancer. (B) Comparison of total immune markers in matched pre-NAC and post-NAC group. X-axis represents the single/combined immune markers. Y-axis indicates the immune infiltrating density that is transformed using Log2 (density +1). NAC, neoadjuvant chemotherapy.

**Figure 3:**
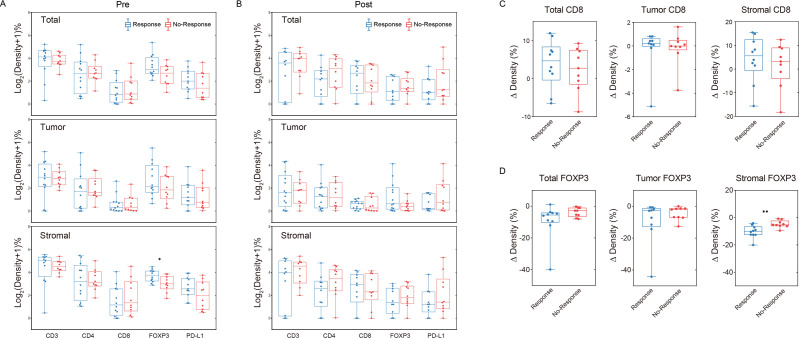
(A) Comparison of immune markers in total, tumor and stromal before NAC between the respond (TRG0-1) and non-respond (TRG2-3) group. (B) Comparison of immune markers in total, tumor and stromal after NAC between the respond (TRG0-1) and non-respond (TRG2-3) group. X-axis represents the single/combined immune markers. Y-axis indicates the immune infiltrating density that is transformed using Log2(densitiy +1). (C) Comparison of the alterations in CD8+ immune cells between the respond (TRG0-1) and non-respond (TRG2-3) group. (D) Comparison of the alterations in FOXP3+ immune cells between the respond (TRG0-1) and non-respond (TRG2-3) group. X-axis represents the respond/no-respond group for NAC, and Y-axis indicates the change of immune infiltrating density after NAC, which is represented by density in post-NAC group minus density in pre-NAC group. NAC, neoadjuvant chemotherapy.

### Multiplex immunofluorescence

Multiplexed tyramide signal amplification (TSA) immunofluorescent staining was performed on pre-NAC and post-NAC GC samples to phenotype and enumerate different tumor-infiltrating T cell populations using the OPAL 6-color fluorescence immunohistochemistry (IHC) Kit (Perkin Elmer, USA). Sections (5 μm thick) were cut from the FFPE blocks of the gastric tumors. The slides were deparaffinized in xylene, rehydrated, and washed before boiling in Tris–EDTA buffer (pH 9) for epitope retrieval. Endogenous peroxidase was blocked using Antibody Diluent/Block (72424205; PerkinElmer, Massachusetts, USA). Primary antibodies to CD3 (ab699, Abcam), CD8 (ab101500, Abcam), CD4 (ab133616, Abcam), FoxP3 (ab20034, Abcam), and PDL1 (SP142, Roche) were incubated for 1 hour at room temperature. Incubation with Opal Ploymer HRP Ms+Rb (2414515; PerkinElmer) was performed for 10 min at 37°C. The slides were then incubated with Opal TSA fluorochromes (Opal540, Opal570, Opal620, Opal650, and Opal690) diluted in amplification buffer for 10 min at RT. The primary and secondary antibody complex was stripped by either microwave treatment with 0.05% ProClin300/Tris–EDTA buffer at pH 9.0. TSA single stain slides were counterstained with DAPI for 5 min and were enclosed in Antifade Mounting Medium (I0052; NobleRyder, Beijing, China). Multiplex TSA IHC was optimized by testing all antibodies individually to test different orders, incubation times, and antibody dilutions. Slides were scanned using the PerkinElmer Vectra (Vectra 3.0.5; PerkinElmer). The ‘tumor mask’ related function of inForm software was used to define the tumor compartment on the sections. A selection of 15 representative original multispectral images were trained to build algorithm (tissue segmentation, cell segmentation, phenotyping tool and positivity score) using inForm software. Then, the algorithm was applied to batch analysis of all the images. More than 10 fields per slide were selected to calculate the percentage of positive cells in all nucleated cells of the tumor nests and tumor stroma. Tonsil samples were used as positive controls for all of the markers.

### TCR variable β-chain sequencing analysis

Immunosequencing of the CDR3 regions of human TCRβ chains was performed using the ImmunoSEQ Assay (Adaptive Biotechnologies). DNA remaining from WES^25^ was amplified in a bias-controlled multiplex PCR to generate the sequencing libraries. Libraries were then sequenced using Illumina MiSeq. Sequencing reads were collapsed and filtered in order to identify and quantitate the absolute abundance of each unique TCRβ CDR3 region for further analysis. In order to estimate the underlying distribution of the entire repertoire, TCR clonal proportion assessment was defined based on the concept of relative species abundance in ecology. Relative abundance refers to the percentage of a specific species of organisms relative to the total number of organisms in the area. The underlying distribution of the repertoire is shown as a stacked barplot by using the immunarch package^26^ in R. In addition, we used D50 and normalized Shannon entropy to define the diversity of TCR. D50 is a recently developed immune diversity assessment indicator that is used to calculate the minimum number of different clone types that are greater than or equal to 50% of the total TCRβ CDR3 sequences.^27^ The normalized Shannon entropy is used to measure the uncertainty of the amount of information and defines the diversity of the TCR repertoire based on the frequency of different TCR types. HNorm=−(Σpi ×log(Pi))/ln(N), N is the number of all TCRs, and pi is obtained by dividing the number of TCR occurrences by the total TCR. In this case, HNorm is between 0 and 1.

### Statistical analysis

Paired Wilcoxon rank-sum test was used to compare the differences of different cell fraction, TCR count, diversity, proportion and tumor mutation burden (TMB) between paired biopsy specimens and post-NAC tumor samples. Spearman correlation analysis was assessed by Fisher’s exact or Cochran-Mantel-Haensel χ^2^ test. Overall survival was calculated using the Kaplan-Meier method, with log-rank test to determine significance of differences. HRs of variables were calculated by univariable Cox regression model, and those having p values up to 0.05 were included in a multivariable Cox regression, combined with iterative backward LR method to identify independent prognostic variables. All statistical tests were two sided at the 5% level of significance. False discovery rate was controlled by applying the explorative Simes (Benjamini-Hochberg) procedure group-wise for each biomarker.^28^ We wrote the article in accordance with the criteria specified in the reporting recommendations for tumor marker prognostic studies (REMARK).^29^ Statistical analyses were performed with SPSS V.23.0 (IBM Corporation) and R (V.4.1.0).

## Results

### Variations of TAICs between NAC and non-NAC group in patients with GC

We first analyzed the difference of TAICs in a large cohort of GC patients, which were divided into two groups: patients who received NAC before surgery (NAC group: n=341) and who did not receive NAC treatment before surgery (non-NAC group, n=1075). The patents’ characteristics are shown in online supplemental table S2). TAICs including pan-T cells (CD3+), T-helper cells (CD4+), regulatory T cells (T-regs (FOXP3+), T-cytotoxic cells (CD8 +GZMB+), B cells (CD20+), natural killer cells (CD57+), and macrophages (CD68+) were detected by IHC on surgical GC samples without or after NAC chemotherapy, respectively. After excluding the spots without more than 50% tumor cells, 308 GC samples after NAC and 1019 treatment naïve GC samples were finally included in the further immune markers analysis.

The association of TAICs with clinicopathological features of GC treated with or without NAC was evaluated. In the treatment-naïve group, infiltrating CD3+, CD4+, and CD8+ T cells and CD20+ B cell were associated with prolonged survival of GC patients (CD3, HR=0.973 (95% CI 0.962 to 0.984), p<0.001; CD4, HR=0.974 (95% CI 0.956 to 0.992), p=0.005; CD8, HR=0.970 (95% CI 0.956 to 0.983), p<0.001; CD20, HR=0.974 (95% CI 0.958 to 0.989), p=0.001) (online supplemental table S3). Multivariate cox survival analysis further confirmed that CD3+ T cells (HR 0.980; 95% CI 0.968 to 0.992; p=0.002;) was independent prognostic factor for survival, together with age, tumor diameter, pTNM stage, Lauren classification and vascular Invasion. Meanwhile, it was found that the infiltration of CD68^+^ macrophages in NAC group was significantly decreased compared with that in the non-NAC group (p<0.001, figure 1A, online supplemental figure S5), while no significant difference observed with respect to other immune markers.

### Association between post-NAC TAICs, clinicopathological parameters, pathological tumor regression, and survival in NAC group

In total, 341 patients were included in NAC cohort (online supplemental table S2). We further compared the infiltration of TAICs between chemotherapy response and non-response cases in 308 residual tumors after NAC. Post-NAC CD8+ T cells, GZMB+ T cell and CD57+ NK cell levels were significantly higher in chemotherapy response group (p=0.029, p=0.017, p=0.033, respectively. figure 1B). CD20+ B cells infiltration were also elevated in the chemotherapy effective cases (p=0.015), while CD68 +macrophages decreased remarkably (p=0.005, figure 1B). In logistic regression univariate analysis, age, vascular invasion, CD20+ B cells, CD68+ macrophages, and CD8+ T cells levels were significantly associated with chemotherapy response (online supplemental table S4). While age remained significant in the multivariate analysis in this GC cohort (online supplemental table S4). Then the survival analysis was performed to determine the association between prognosis and clinicopathological factors including TAICs. The median survival time of this cohort was 32.3 months (range:20.4–44.2). Patients with a higher infiltration of CD3+ and CD8+ T cells had a longer overall survival (OS) than patients with a lower level (CD3: HR 0.982, 95% CI 0.965 to 0.999, p=0.043; CD8: HR 0.972, 95% CI 0.953 to 0.991, p=0.004, table 1). Multivariate cox survival analysis showed that CD8+ T cell levels remained as an independent factor (HR 0.967; 95% CI 0.946 to 0.989; p=0.004), together with pTNM stage, and differentiation (table 1).

**Table 1:** Univariate and multivariate survival analysis in GC NAC cohort

Characteristics		Number		Univariate		Multivariate	
	Class	Total	Event (OS)	HR (95% CI)	P value	HR (95% CI)	P value
Age				1.006 (0.991 to 1.020)	0.450		
Histology	Adenocarcinoma	283	161	1		1	
	Signet-ring cell carcinoma (SRCC)	19	15	1.772 (1.043 to 3.009)	0.034	1.993 (0.968 to 4.105)	0.061
Differentiation							
	Poor	135	91	1		1	
	Well/moderate	144	74	0.632 (0.465 to 0.859)	0.003	0.636 (0.439 to 0.922)	0.017
Tumor diameter							
	<5 cm	151	72	1			
	≥5 cm	137	97	1.870 (1.378 to 2.538)	<0.001		
Vascular invasion							
	Negative	174	80	1			
	Positive	123	93	2.605 (1.926 to 3.523)	<0.001		
NAC response							
	Responders	28	10	1			
	Non-responders	276	167	2.116 (1.118 to 4.007)	0.021		
pTNM							
	I	23	1	1		1	
	II	95	30	7.711 (1.051 to 56.543)	0.045	3.627 (0.488 to 26.968)	0.208
	III	127	99	30.607 (4.263 to 219.727)	0.001	12.691 (1.760 to 91.496)	0.012
	IV	43	39	47.637 (6.530 to 347.492)	<0.001	17.340 (2.346 to 128.150)	0.005
CD3				0.982 (0.965 to 0.999)	0.043		
CD8				0.972 (0.953 to 0.991)	0.004	0.967 (0.946 to 0.989)	0.004
CD4				0.984 (0.957 to 1.012)	0.255		
FOXP3				1.004 (0.906 to 1.113)	0.944		
Granzyme B				0.943 (0.860 to 1.035)	0.220		
CD20				1.004 (0.982 to 1.027)	0.725		
CD68				0.997 (0.963 to 1.032)	0.873		
CD57				1.009 (0.987 to 1.032)	0.439		

GC, gastric cancer; NAC, neoadjuvant chemotherapy.

### TIL variation before and after NAC and the relationship with pathological tumor response

To further compare the NAC effect on immune microenvironment, a panel of multiplex immunofluorescence staining (CD3, CD4, CD8, Foxp3, and PDL1; figure 2A) were performed in paired samples before and after chemotherapy in another independent cohort of 30 patients with GC including 15 response cases and 15 non-response cases (online supplemental table S5).

Compared with biopsy specimens, Foxp3+ T reg cells proportion significantly decreased in both epithelial and stromal compartments (p<0.001, figure 2B, online supplemental figure S6A, B). Infiltration of T-reg cells (FoxP3+/CD3+) expressing PDL1 were also reduced significantly after chemotherapy (p=0.026, figure 2B, online supplemental figure S6A, B). Also, pre-NAC Foxp3 T-reg cells level was much richer in the response group (p=0.036, figure 3A) and further decreased more significantly in the stromal compartment after chemotherapy (p=0.008, figure 3D). The response predictive efficacy of stromal Foxp3+ T reg cells density before NAC was evaluated by ROC analysis, the area under curve achieved 0.773 (95% CI 0.571 to 0.975, online supplemental figure S6C).

Besides, CD8+ cytotoxic T lymphocytes were significantly elevated in the surgical samples after chemotherapy than in the biopsy specimens (p=0.029, figure 2B). Further subgroup analysis revealed that CD8+ cytotoxic T cells elevation was more significant in the group treated with XELOX regimen (p=0.016, online supplemental figure S7). Compared with the non-response group, CD8+ cytotoxic T cells elevation was more obvious in the response group (online supplemental figure S8). We also observed that CD3+ T cells infiltration significantly downregulated after chemotherapy in the epithelial and stromal compartments respectively, together with CD4+ T helper cells in the epithelial areas (online supplemental figure S6A, B).

### TCR β-chain sequencing between pre-NAC and post-NAC tumor samples

**Figure 4:**
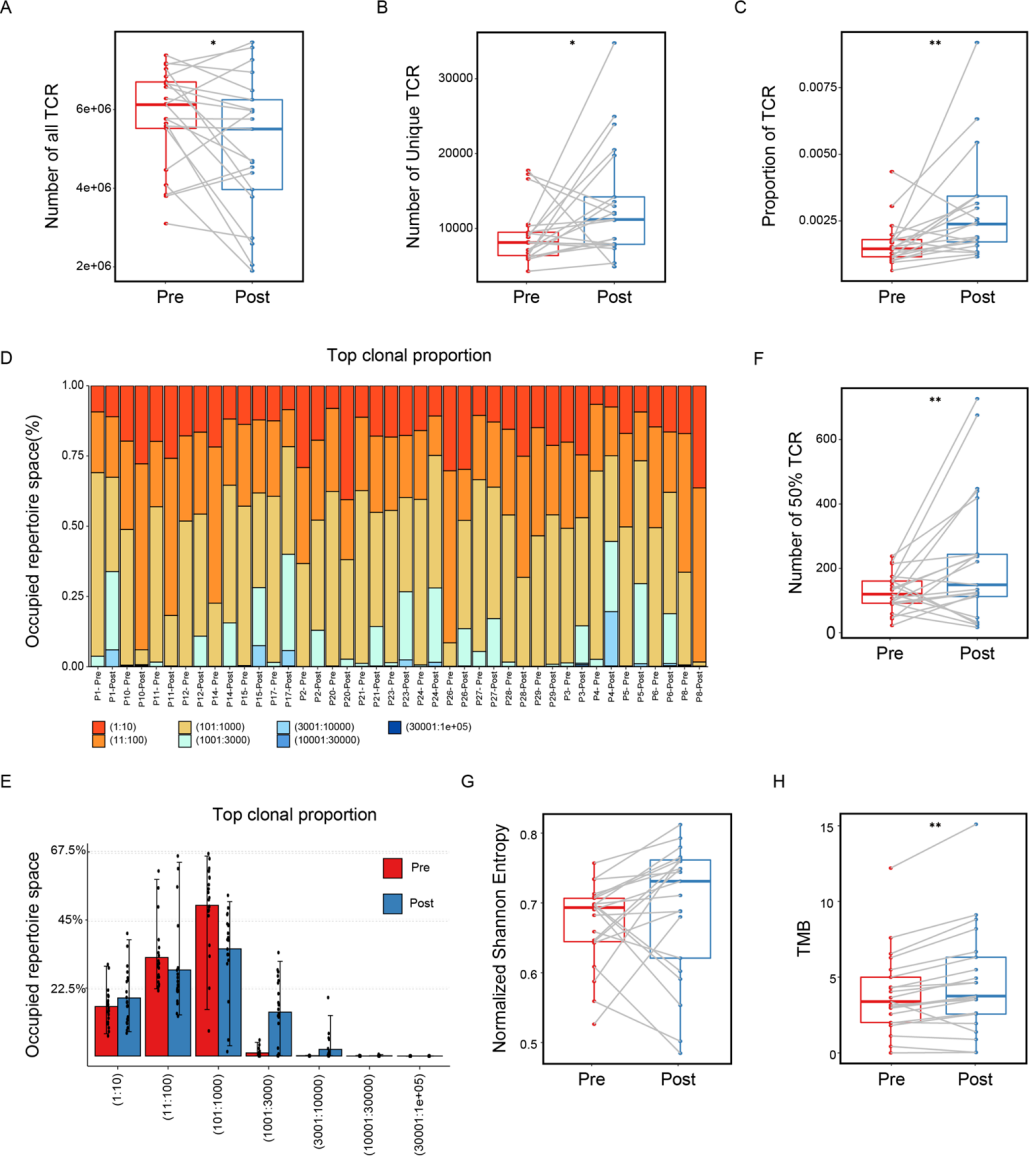
T cell receptor repertoire analysis of preneoadjuvant and postneoadjuvant chemotherapy-treated gastric cancer. Comparison of the number of total TCR (A) and unique TCR (B) and the proportion of unique TCR (C) in patients treated with and without NAC. Estimation relative abundance for the groups of top TCR clonotypes in TCR repertoire of all patients (D) and of paired pre-NAC and post-NAC group (E). The D50 index (F) and normalized Shannon entropy (G) of the TCR repertoire before and after NAC for each patient. (H) Comparison of the number of tumor mutation burdens (TMBs) pre-NAC and post-NAC for each patient. NAC, neoadjuvant chemotherapy.

Considering the increased CD8+ T cells infiltration after NAC, we further characterize the T cell repertoire through sequencing the CDR3 region of TCR β-chain in the paired pre-NAC and post-NAC samples mentioned previously. The statistical analysis of the TCR sequencing results of all patients are shown in figure 4. We observed significantly lower infiltrating TCR count but higher number of unique TCR clones in post-NAC samples compared with pre-NAC samples (figure 4A.B). As a result, the proportion of TCR was significantly higher in post-NAC group, suggesting that the diversity of T cells was increased after chemotherapy (figure 4C). The underlying distribution of the TCR repertoire presented approximately as power law distribution, a few TCR account for most of the clones of the whole repertoire. The comparison of pre-NAC and post-NAC TCR distribution indicating that the clonal expanded T cells, which contribute to the increased diversity of T cells in post-NAC samples, were eliminated after chemotherapy (figure 4D, E). We then measured the D50 index and normalized Shannon entropy (see Methods) of all patients to characterize the normalized diversity of TCR repertoire. D50 index after NAC was significantly higher than that before NAC, meanwhile, the median of normalized Shannon entropy was also higher than that before NAC (figure 4F, G), which were consistent with the previous results. In total, these results suggesting that NAC has changed the overall distribution of tumor infiltrated T cells. Besides, the number of TMB increased markedly after chemotherapy (figure 4H), which may potentially explain the increased diversity of T cells after treatment.

**Figure 5:**
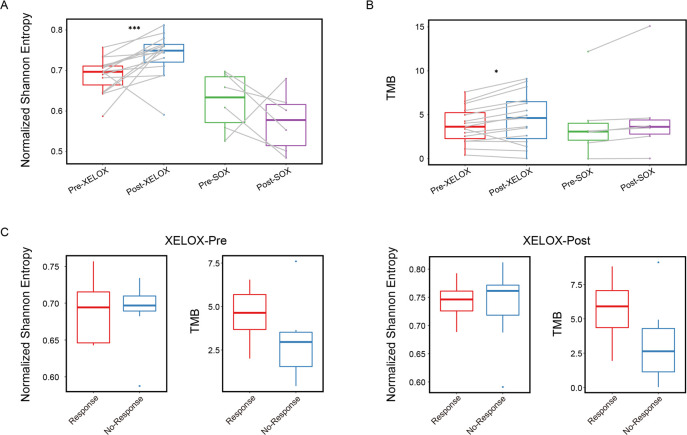
Analysis of different chemotherapy regimens. Comparison of the normalized Shannon entropy (A) and tumor mutation burdens (B) of the TCR repertoire pre-NAC and post-NAC for each patient in different regimens. (C) Comparison of the normalized Shannon entropy and tumor mutation burdens of the TCR repertoire in matched pre-NAC and post-NAC group for patients accepted XELOX regimen by effect.

Furthermore, in order to examine the impact of different chemotherapy regimen, we grouped all patients into XELOX and SOX subset and analyzed the diversity of TCR repertoire. Interestingly, the normalized Shannon entropy of the patients with XELOX regimen increased significantly after NAC, while SOX group decreased in the diversity of the TCR repertoire (figure 5A). We speculate that this phenomenon might be due to the mechanism of different chemotherapy regimens. Different chemotherapy regimens showed no effect on the change of the TMB between pre-NAC and post-NAC group (figure 5B). Combined with the efficacy of XELOX regimen, we observed that the diversity of ineffective group after chemotherapy was slightly higher than that of the effective group, and the number of TMB in the effective group increased, although the results were not significant due to the small sample size (figure 5C). In line with previous studies,^30^ patients with high TMB had a higher probability of clinical benefit in treatment. We next examined the relationship between the number of neoantigen and TMB and the normalized Shannon entropy. The number of tumor neoantigen was positively correlated with the number of TMB but showed no correlation with the normalized Shannon entropy before or after treatment (online supplemental figure S9), suggesting that NAC has impact on both tumor cells and the infiltrated T cells.

## Discussion

NAC has been increasingly used to improve the curative surgical resection and decrease the risk of micrometastasis, thus prolonging the survival of patients with advanced GC. In this era of cancer immunotherapy, accumulating evidence has shown that the conventional chemotherapy could have immunoregulatory effects. However, information is lacking on the impact of NAC on the local immune microenvironment in GC. This study is the first to use multiplex IHC and TCR sequencing on matched pre-NAC and post-NAC GC samples to analyze and compare functionally divergent tumor-infiltrating T cell subpopulations and clonal expansion. The prognostic and predictive values of post-NAC TAICs were also calculated in a cohort of 341 NAC GC patients.

Previous studies have demonstrated a specific increase in CD8+ TILs and a decrease in FOXP3+, CD4+, CD20+ and CD68+ immune cells after NAC in breast cancer.^31–35^ The prevailing view is that recruitment of CD8+ cytotoxic T cells post-NAC is associated with a better outcome and that an immunological profile combining low/absence of immunosuppressive FOXP3 cells and high number of activated CD8+ T cells in residual breast tumors post-NAC is associated with improved survival,^36^ highlighting the importance of the balance between cytotoxic and suppressive T cells.

T-regs are a kind of immunosuppressive T-lymphocytes that play a major role in immune escape and suppressing antitumor immune response, thus promoting tumor growth and invasion. This study demonstrated NAC decreased Foxp3+ T regs in stroma and correlated with better response, suggesting that immune suppression of patients with advanced GC was significantly relieved after NAC. T-regs exert suppressive activities on effector cells, partly via the upregulated the inhibitory immune checkpoint molecules expression.^37^ This study displayed that infiltration of T-reg cells expressing PDL1 were also significantly reduced after NAC. It is consistent with previous studies reported that T-regs are sensitive to chemotherapy, and the antitumor activity of chemotherapy may be mediated by depletion and suppression of T-regs and lower number of intratumoral Foxp3 T-regs are associated with favorable prognosis in breast cancer and GC.^38 39^

Meanwhile, our results revealed NAC increased CD8+ cytotoxic T cells infiltration in the TMEs, and the T cell receptor diversity was also observed as elevated, suggesting that chemotherapy can increase T cell expansion and priming into the TME and enhance antitumor immunity. Besides, the XELOX regimen has more potential than SOX regimen to induce immunogenic tumor cells death and release tumor antigens that are taken up by the surrounding immune cells, resulting in CD8+ T cells activation and expansion. Difference between XELOX and SOX regimen is mainly based on the5-fluorouracil (5-FU) precursor drugs, XELOX regimen containing capecitabine and SOX with S1. Possible explanation could be that capecitabine is more likely to increase efficacy by increasing the concentration of 5-FU in tumor tissues, while S1 increasing the blood concentration of 5-FU. Besides, it has been reported that platinum increased intra-tumor CD4+ and CD8+ T cell trafficking in esophageal cancer.^40^ Carboplatin and paclitaxel have been reported to synergistically augment tumor-specific CD8+ cytotoxic responses in both mouse models and patients.^41^ Thus, potential combinations including T cell activating agents (TLR9, STING, and IL-10 agonists) may represent promising approaches to further augment antitumor response in GC patients.

Furthermore, we found the compartmental localization of FOXP3+ T reg cells might influence the impact of tumor immunity. It is reported that quantity of memory/regulatory T cells (CD45R0+FOXP3+) was significantly lower in the stromal compartment in NAC group than in non-NAC patients, suggesting that chemotherapy can regulate phenotype by tumor tissue compartment.^23^ Our results showed FOXP3+ T reg cells infiltration in the stromal area were significantly higher in response group and could be a predictive factor for chemotherapy efficacy. However, there is currently no standardized approach to evaluate TAICs in GC, the difference between intraepithelial/intrastromal/intratumoral/peritumoral TAICs is vaguely defined. Based on the studies of breast cancer in which the definition of TILs in different compartment was well establish by the international TILs Working Group, stromal TILs have emerged as a significant prognostic marker than intraepithelial TILs.^42 43^

In addition, the present study showed that NAC could impact infiltrating immune components tumor cells. Increased number of TMB was observed after NAC, and neoantigen was positively correlate with TMB rather than diversity of TCR. To gain more mechanistic insights, we further analyzed transcriptional data of another NAC cohort (PKUCH NAC cohort) from our previous published study.^44^ Through Single-sample gene set enrichment analysis (ssGSEA) algorithm (online supplemental method), we compared immune components between 14 paired NAC samples and validated the change of immune infiltration after NAC (online supplemental figure S10A). Meanwhile, we found that cancer-associated signaling pathways, such as hypoxia pathway, MAPK, Wnt and STAT3 signaling pathways, were also downregulated following NAC (online supplemental figure S10B, C). Correlation analysis showed that several classical oncogenic pathways were positively correlated with T-reg infiltration (online supplemental figure S10C), which were reported to be involved in T-reg cells infiltration in cancer.^45 46^ In fact, a number of latest researches explored the genomic and transcriptional alterations in cancer by analyzing pre-NAC and post-NAC tissues.^47 48^ Some of those studies found the immune-related genes or pathways altered by NAC. Therefore, we considered that NAC possibly reorganized the interaction network between tumor and TME, which reversed immune suppressive TME and inhibited tumor progression.

As a retrospective study, our study has several limitations, which should be taken into consideration when interpreting the data. First, the small number of paired pre-NAC and post-NAC samples provide limited statistical power necessary to differentiate the TME between responders and non-responders. Future studies should take the histological features, clinical stage and molecular subtype into account. Second, this study is mainly focused on the T cells, further researches are required to increase the power of this preliminary findings and expand the detection on tumor cells, dendritic cells, macrophages and other myeloid-derived cells together with checkpoint expression in GC TME to unravel the interplay with T cells. Third, considering the heterogeneity of the GC tissues, further studies should take the spatial distribution of TAICs into account, such as tumor center, tumor periphery, cancer nest and stroma. Also, the biopsy tissues may only reveal focal information, which might not sufficient to represent the whole tumor from surgical samples. However, several studies have reported the consistence of TILs evaluation between biopsies and resected specimens in breast cancer.^49 50^

In a summary, this study, to our knowledge, is the biggest study describing the impact of NAC on immune microenvironment. The results have shown that NAC has the capacity to alter the infiltration and subtypes of immune cells by significantly decreasing FOXP3+ T reg cells, increasing CD8+ cytotoxic cells and TCR diversity. Besides, CD8+ T cells levels post-NAC significantly predict prognosis. These data indicate the heterogeneity in both tumor and host factors in GC. NAC may regulate the equilibrium of immune subtypes in favor of immune activation and improved outcome in a subset of GC patients. In the future, the personalized approach based on the immune subpopulations and expression of coregulatory molecules would be better identified after NAC to enhance efficacy of combinational therapy and final improve outcome. A number of immune therapies are in development targeting various coregulatory molecules on T cells and macrophages. Incorporating information regarding the immune related profile after NAC may lead to a more effective and personalized immune strategy in GC.

## Data Availability

The data are available from the corresponding author on reasonable request.
