# Supplementary material for: Effect of neoadjuvant chemotherapy on the immune microenvironment in gastric cancer as determined by multiplex immunofluorescence and T cell receptor repertoire analysis
Source: J Immunother Cancer. 2022 Mar 31;10(3):e003984. doi: 10.1136/jitc-2021-003984 (PMC8971786; doi:10.1136/jitc-2021-003984)
Supplement: Supplementary data [file jitc-2021-003984supp001.pdf]

Supplementary Table 1. Antibodies

Antibody	Cat. Number	Company	Application
CD3	NCL-CD3-565	Novo	IHC
CD4	4B12	DAKO	IHC
CD8	CRM311C	Biocare	IHC
CD68	Z2071	Zeta	IHC
CD274	SP142	Roche	IHC
FOXP3	98377	Cell Signaling Technology	IHC
GZMB	GrB-7	DAKO	IHC
CD20	L26	DAKO	IHC
PDL1	13684	Cell Signaling Technology	IHC

Supplementary Table S2. Characteristics of GC patients who received neoadjuvant chemotherapy (NAC) or not(non-NAC)

Characteristics	Class	NAC group (n=341)	non-NAC group (n=1075)	*p*
Age		59 (52-66)	60 (52-69)	0.024
Gender				0.023
	Male	269	781	
	Female	72	294	
Histology				0.245
	Ade	317	972	
	Ring	21	87	
Differentiation				0.746
	poor	155	498	
	Well/moderate	155	521	
Diam				0.897
	0	173	561	
	1	143	473	
Vascular Invasion				<0.001
	No	202	489	
	Yes	130	570	
Lauren Classification				0.006
	D	72	260	
	I	202	589	
	M	35	190	
cTNM				<0.001
	I	24	119	
	II	98	355	
	III	139	433	
	IV	46	68	
EBER				0.105
	Negative	296	958	
	Positive	18	36	
MVD				1.000
	<25%	75	242	
	25-75%	148	478	
	>75%	75	242	

Supplementary Table S3 Univariate and multivariate survival analysis in GC treatment naïve cohort

Characteristics	Class	Number		Univariate		Multivariate	
		Total	Event (OS)	HR (95% CI)	*p*	HR (95% CI)	*p*
Age				1.019(1.011-1.028)	<0.001	1.021 (1.012-1.031)	<0.001
Differentiation	poor	452	240	1			
	Well/moderate	488	216	0.820(0.683-0.986)	0.035		
Tumor Diameter	<5cm	511	194	1		1	
	>=5cm	447	266	2.003(1.664-2.412)	<0.001	1.259 (1.023-1.551)	0.030
Vascular Invasion	negative	442	157	1		1	
	positive	534	313	2.176(1.796-2.637)	<0.001	1.362 (1.090-1.702)	0.007
Lauren Classification	Diffused	237	136	1		1	
	Intestinal	554	241	0.692(0.560-0.853)	0.001	0.679 (0.535-0.862)	0.001
	Mixed	179	92	0.875(0.672-1.140)	0.323	0.856 (0.641-1.142)	0.291
pTNM stage	I	112	13	1		1	
	II	273	89	3.102(1.733-5.552)	<0.001	3.197 (1.594-6.409)	0.001
	III	508	307	7.957(4.566-13.868)	<0.001	6.936 (3.505-13.727)	<0.001
	IV	69	60	24.747(13.538-45.234)	<0.001	21.717(10.426-45.235)	<0.001
CD3				0.973(0.962-0.984)	<0.001	0.980 (0.968-0.992)	0.002
CD4				0.974(0.956-0.992)	0.005	0.983 (0.963-1.003)	0.099
CD8				0.970(0.956-0.983)	<0.001		
CD20				0.974(0.958-0.989)	0.001		
FOXP3				1.013(0.987-1.039)	0.343		
CD57				0.993(0.979-1.007)	0.302		
CD68				1.008(0.986-1.031)	0.459		

Supplementary Table S4. Correlation analysis of TILs with clinical features in NAC cohort

		Number		Univariate		Multivariate	
**Characteristics**	**Class**	**Total**	**responders**	**OR (95% CI)**	***p***	**OR (95% CI)**	***p***
Age				0.953 (0.920-0.988)	0.008	0.913(0.854-0.977)	0.008
Differentiation	poor	137	15	1			
	Well/moderate	146	11	0.663 (0.293-1.498)	0.323		
Tumor Diameter	<5cm	154	17	1			
	>=5cm	138	8	0.496 (0.207-1.188)	0.116		
Vascular Invasion	negative	177	24	1			
	positive	124	5	0.268 (0.099-0.723)	0.009		
Lauren Classification	Diffused	63	6	1			
	Intestinal	197	13	0.671 (0.224-1.846)	0.440		
	Mixed	33	2	0.613 (0.117-3.220)	0.563		
cTNM stage	I	17	4	1			
	II	89	11	0.458 (0.127-1.659)	0.235		
	III	128	10	0.275 (0.076-1.004)	0.051		
	IV	42	1	0.079 (0.008-0.774)	0.029		
CD3				0.999 (0.950-1.051)	0.979		
CD8				1.039 (1.003-1.078)	0.036	~~1.091(1.023-1.163)~~	~~0.008~~
CD4				1.010 (0.947-1.077)	0.769		
CD20				1.161 (1.025-1.314)	0.019		
CD57				1.060 (0.989-1.137)	0.101	~~1.048 (0.980-1.121)~~	~~0.174~~
FOXP3				0.996 (0.865-1.148)	0.961		
GranzymeB				1.059 (0.905-1.239)	0.474		
CD68				0.612 (0.403-0.930)	0.021	~~0.736 (0.585-0.925)~~	~~0.009~~

Supplementary Table S5. Paired NAC patients characteristics.

Sample ID	Response	Age	Lauren	TRG	ypTNM	Chemotherapy Regimens	Cycles	TMB
P17T	Yes	63	Mixed	0	0	XELOX	3	5.49342
P19T	Yes	52	Diffuse	0	I	XELOX	3	0.06579
P1T	Yes	69	Mixed	0	0	XELOX	3	2.56579
P23T	Yes	62	Intestinal	0	0	XELOX	4	4.04605
P30T	Yes	61	Mixed	0	0	XELOX	3	5.92105
P10T	Yes	52	Mixed	1	II	SOX	4	4.34211
P11T	Yes	54	Mixed	1	III	SOX	3	0
P12T	Yes	58	Intestinal	1	II	XELOX	3	6.28289
P16T	Yes	67	Diffuse	1	I	SOX	4	7.10526
P18T	Yes	36	Intestinal	1	III	SOX	3	0.72368
P20T	Yes	55	Mixed	1	I	SOX	4	3.15789
P21T	Yes	76	Diffuse	1	I	XELOX+H	2	4.27632
P22T	Yes	70	Intestinal	1	I	XELOX	3	30.4276
P29T	Yes	63	Intestinal	1	I	XELOX	3	5
P28T	No	61	Mixed	2	III	XELOX	3	0.42763
P13T	No	57	Diffuse	3	III	SOX	3	5.03289
P14T	No	54	Intestinal	3	II	XELOX	3	3.65132
P15T	No	59	Mixed	3	II	XELOX	3	1.11842
P24T	No	57	Mixed	3	III	XELOX	3	3.38816
P25T	No	45	Mixed	3	III	SOX	2	0
P26T	No	61	Mixed	3	III	SOX	4	3.02632
P27T	Yes	75	Mixed	3	II	XELOX	3	6.54605
P2T	Yes	43	Mixed	3	I	XELOX	3	2.00658
P3T	No	53	NA	3	III	SOX	4	1.80921
P4T	No	61	Intestinal	3	II	XELOX	3	7.59868
P5T	No	65	Intestinal	3	II	XELOX	3	1.97368
P6T	No	64	Intestinal	3	II	XELOX	3	2.96053
P7T	No	63	Mixed	3	III	XELOX	3	5.72368
P8T	No	58	Intestinal	3	III	SOX	3	12.2039
P9T	No	58	Mixed	3	I	XELOX	3	0.78947

Figure S1.

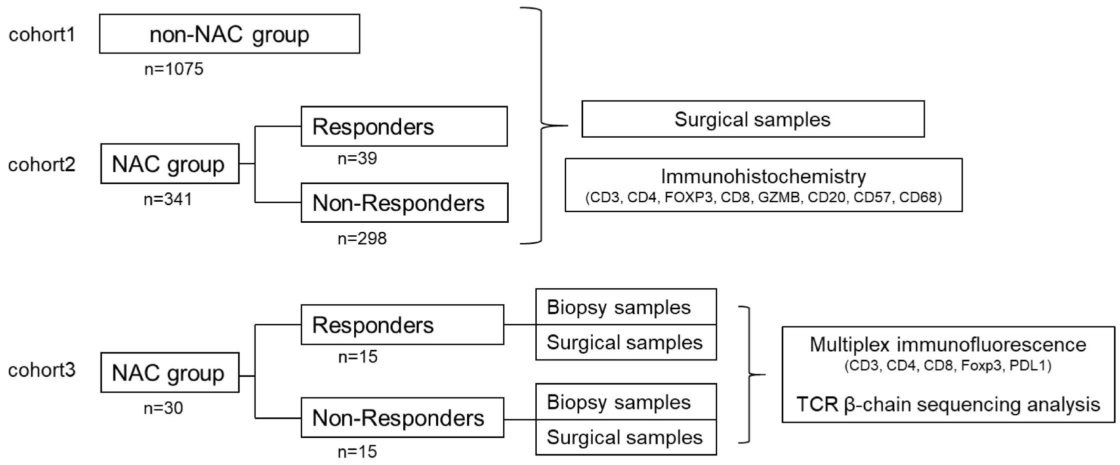

Figure S1. The flow chart of this study.

Figure S2.

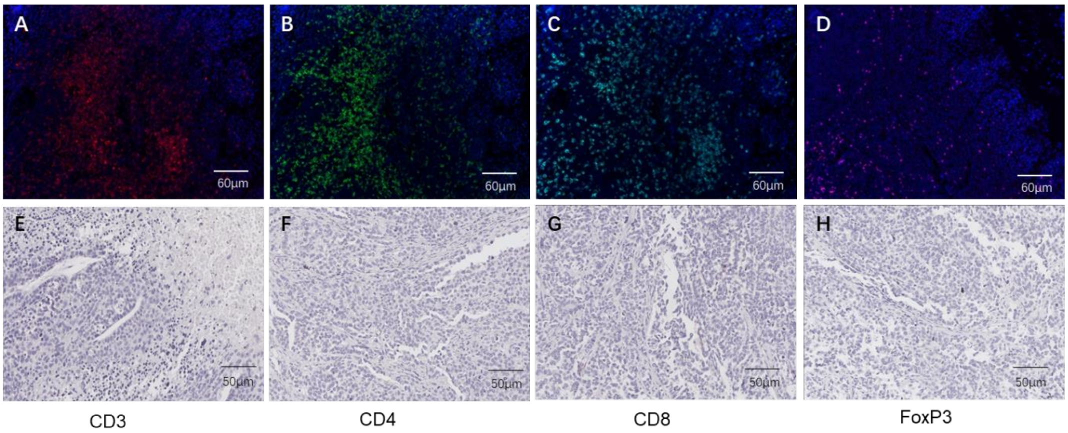

Figure S2. Validation of antibodies of T cell markers(CD3, CD4, CD8, FoxP3). T cells were stained with corresponding antibodies using tonsil tissues as positive control (A,B,C,D) and gastric cancer cell line (BGC823) derived tumor tissues from nude mice as negative control (E,F,G,H).

Figure S3.

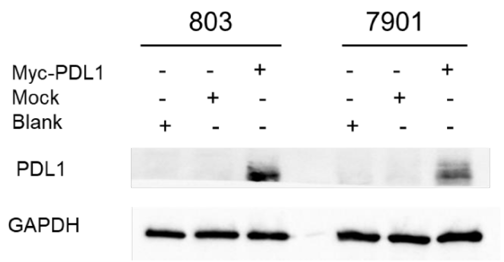

Figure S3. Validation of the specificity of PDL1 antibody. BGC803 and SGC7901 cells were transfected with pcDNA3.0-myc-PDL1 or mock. Overexpression of PDL1 could be detected using antibody SP142.

Figure S4.

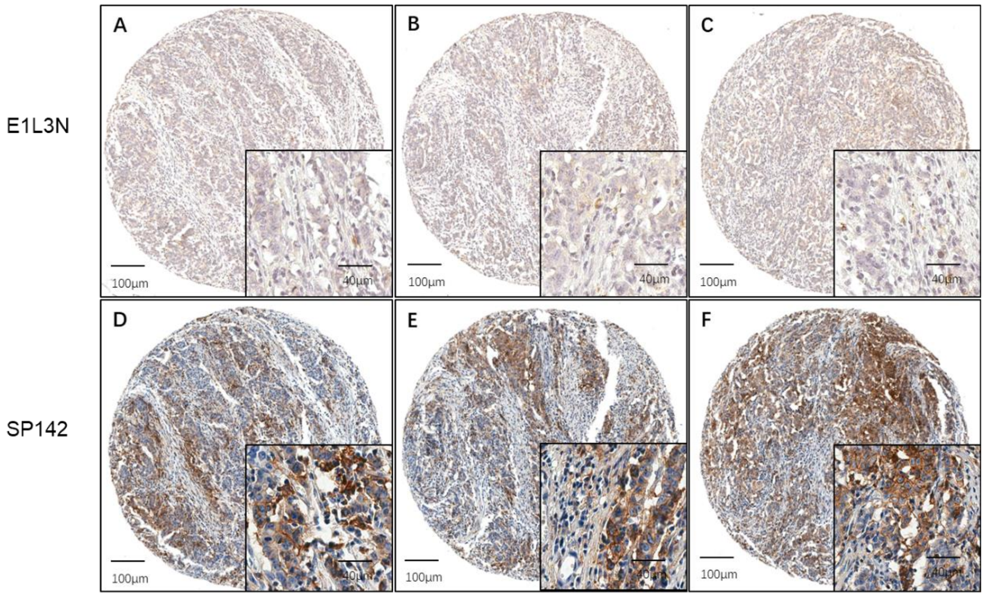

Figure S4. Expression of PDL1 in the identical tumor tissues using SP142 and E1L3N antibody. Spots A, B and C were from the same patient, which showed the same location as spot D, E and F, respectively.

Figure S5.

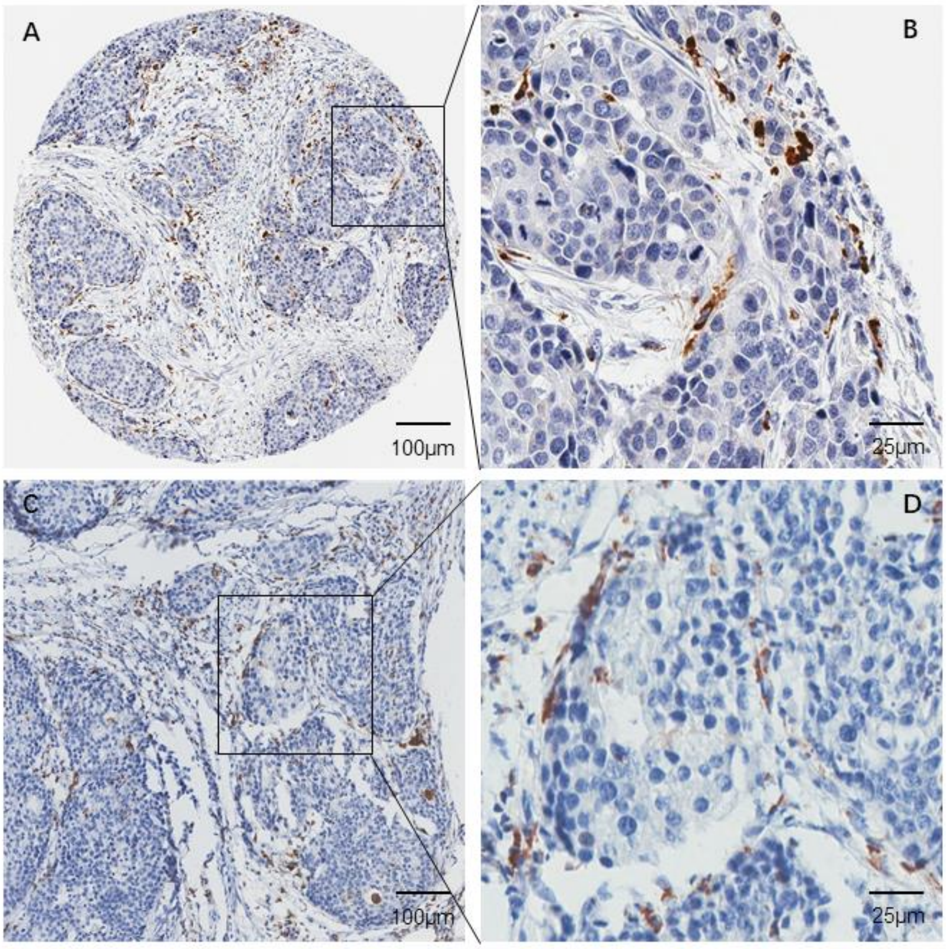

Figure S5. Representative CD68 staining in GC tissue microarray (A, B) and whole slides (C, D) from the same patient.

Figure S6.

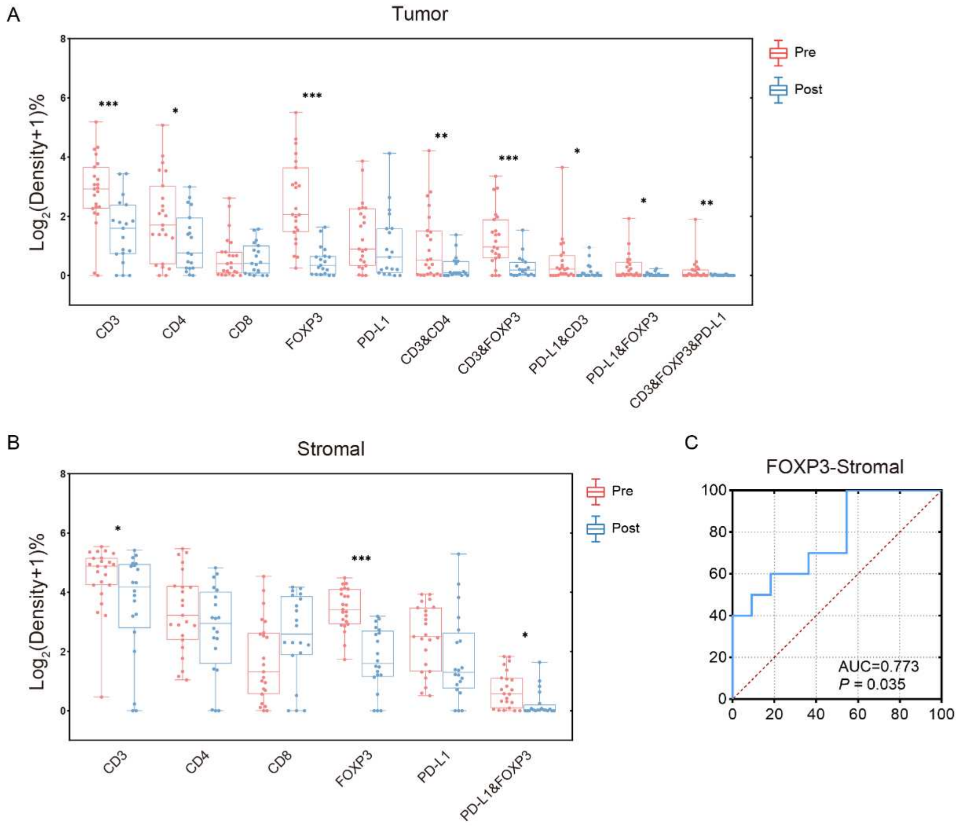

Figure S6. A. Comparison of tumor immune markers in matched pre-and post-NAC group. B. Comparison of stromal immune markers in matched pre-and post-NAC group. X-axis represents the single/combined immune markers. Y-axis indicates the immune infiltrating density that is transformed using Log2(densitiy+1). C. ROC curves for the diagnostic accuracy of stromal FOXP3+ immune cells in predicting the response of NAC.

Figure S7.

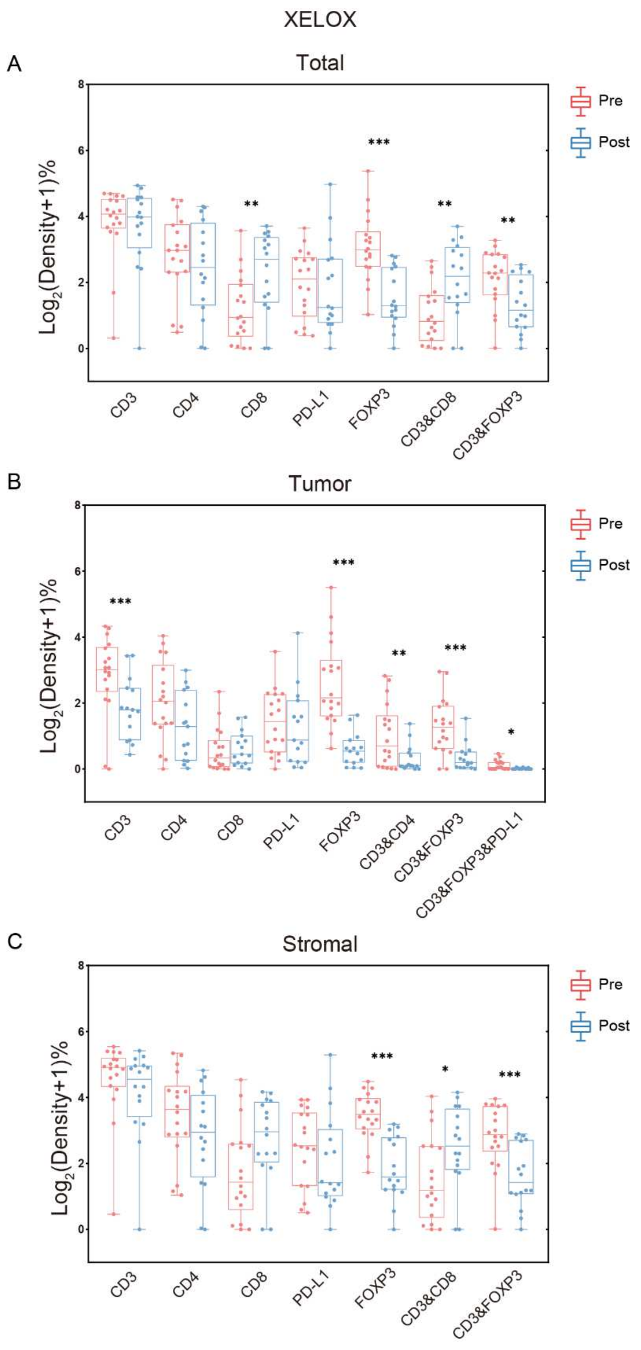

Figure S7. A. Comparison of total immune markers in matched pre-and post-NAC group for patients accepted XELOX regimen. B. Comparison of tumor immune markers in matched pre-and post-NAC group for patients accepted XELOX regimen. C. Comparison of stromal immune markers in matched pre-and post-NAC group for patients accepted XELOX regimen. X-axis represents the single/combined immune markers. Y-axis indicates the immune infiltrating density that is transformed using Log2(densitiy+1).

Figure S8.

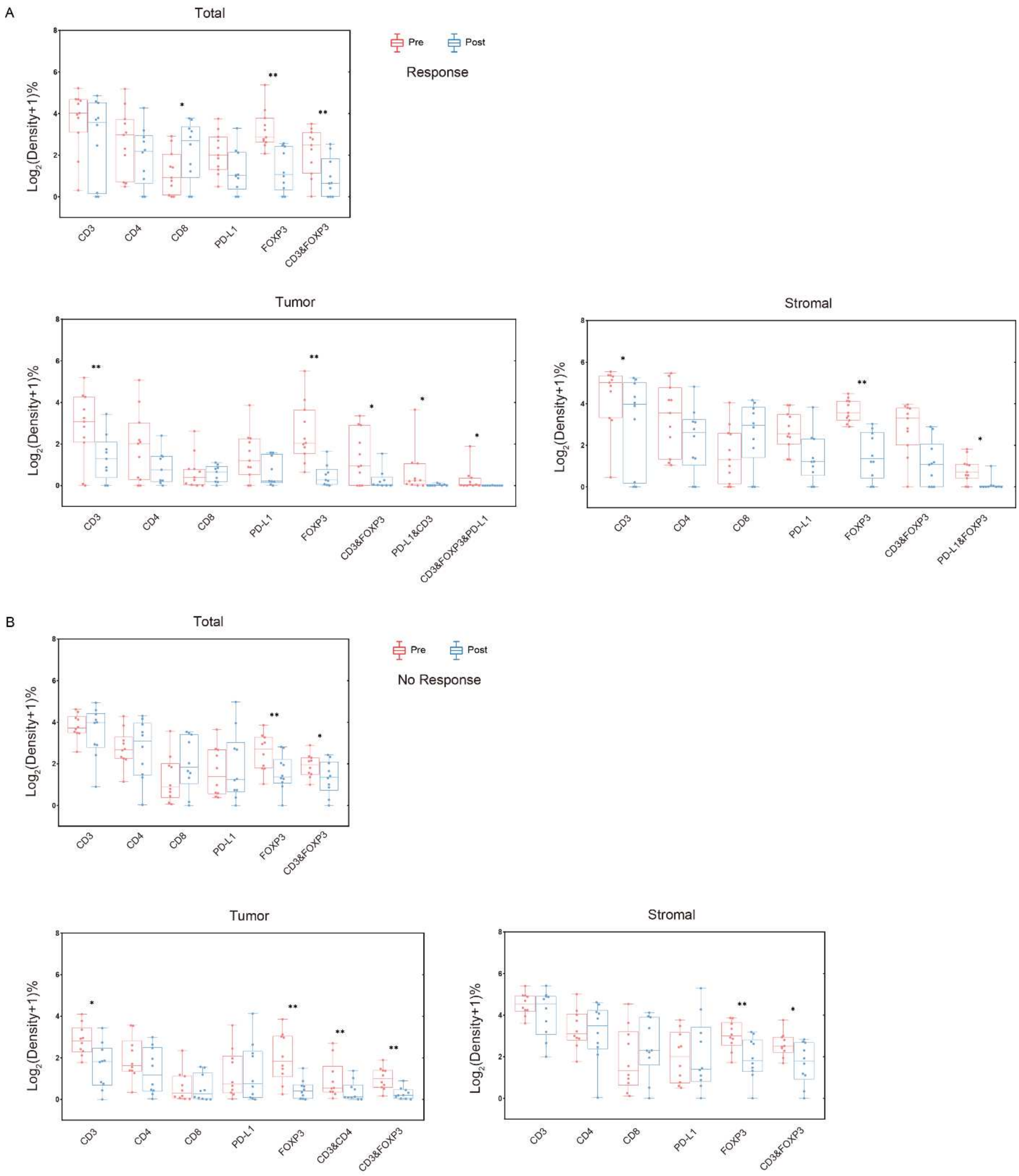

Figure S8. A. Comparison of immune markers in matched pre-and post-NAC group for TRG 0-1 patients. B. Comparison of immune markers in matched pre-and post-NAC group for TRG 2-3 patients. X-axis represents the single/combined immune markers. Y-axis indicates the immune infiltrating density that is transformed using Log2(densitiy+1).

Figure S9.

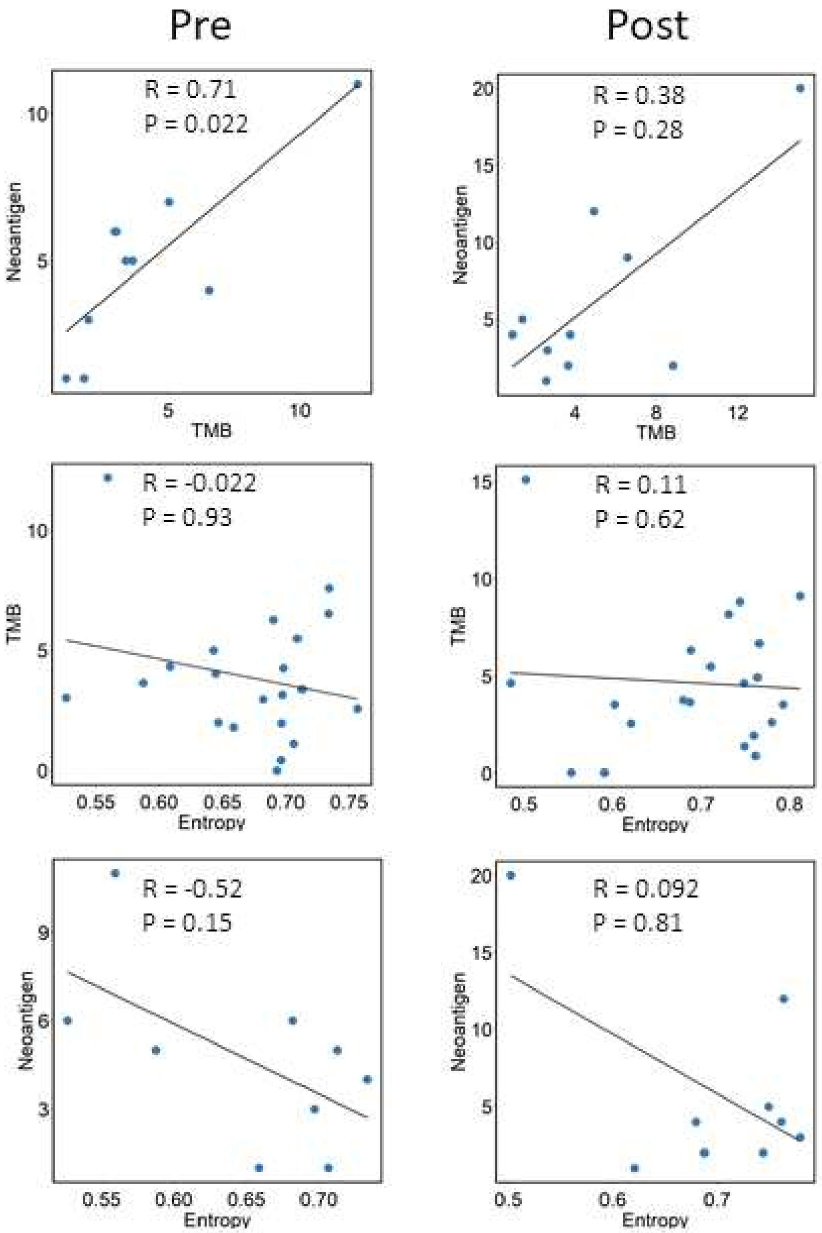

Figure S9. The correlation between the number of neoantigen, the number of TMB and normalized Shannon entropy pre-and post-NAC for each patient.

Figure S10.

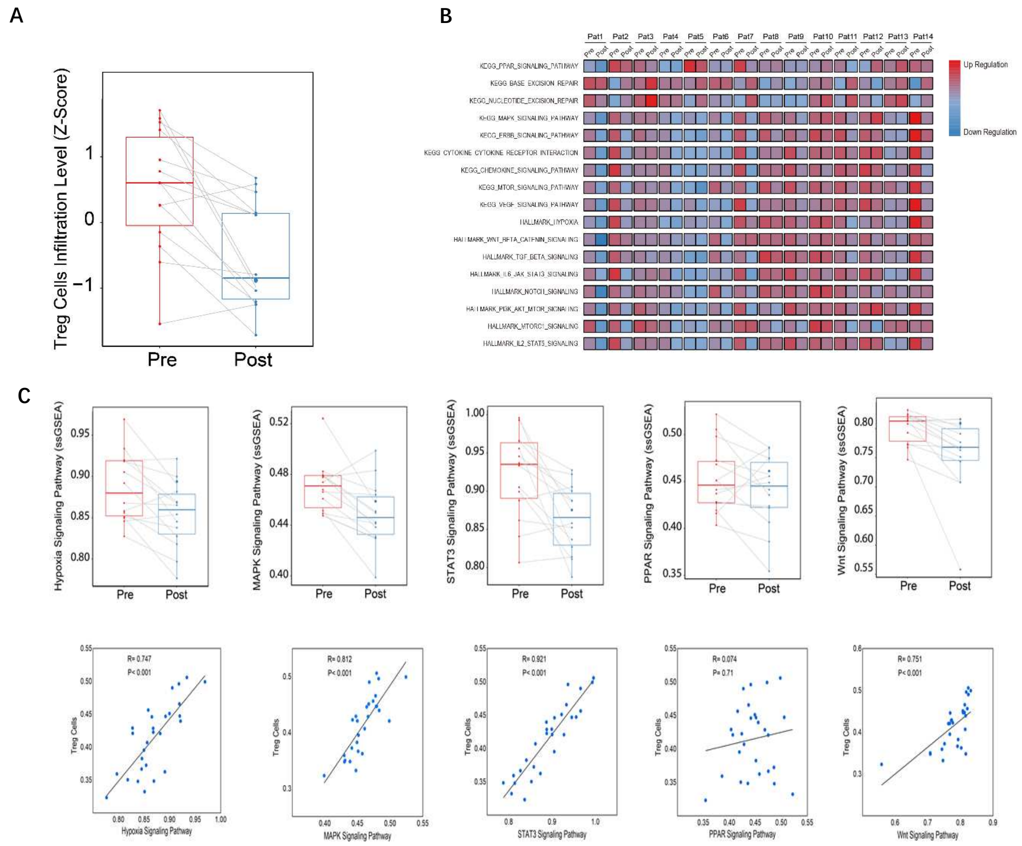

Figure S10. A. Comparison of T-reg cells infiltration in matched pre- and post-NAC samples of PKUCH NAC cohort. The infiltration of T-reg cells was estimated by ssGSEA algorithm and normalized via Z-Score. B. Multiple oncogenic signaling pathways were downregulated after NAC in PKUCH NAC cohort. C. The correlation between the infiltration of T-reg cells and oncogenic signaling pathways.

Supplementary Method

Single-sample gene set enrichment analysis (ssGSEA)

One of our previous studies explored the molecular features of GC in another NAC cohort (PKUCH NAC cohort)[1], which comprised 14 paired pre- and post- NAC samples. Based on transcriptional sequence data from PKUCH NAC cohort, we investigated the impact of NAC on tumor biological signaling pathways using the single-sample gene set enrichment analysis (ssGSEA) algorithm ("GSVA" R packages). The gene set of regulatory T cell was obtained from the study of Charoentong[2]. The hallmark gene sets were downloaded from the Molecular Signatures Database (MSigDB)[3].

1. Li Z, Gao X, Peng X, et al. Multi-omics characterization of molecular features of gastric cancer correlated with response to neoadjuvant chemotherapy. *Sci Adv* 2020;6(9):eaay4211. doi: 10.1126/sciadv.aay4211 [published Online First: 2020/03/07]
2. Charoentong P, Finotello F, Angelova M, et al. Pan-cancer Immunogenomic Analyses Reveal Genotype-Immunophenotype Relationships and Predictors of Response to Checkpoint Blockade. *Cell Rep* 2017;18(1):248-62. doi: 10.1016/j.celrep.2016.12.019 [published Online First: 2017/01/05]
3. Liberzon A, Subramanian A, Pinchback R, et al. Molecular signatures database (MSigDB) 3.0. *Bioinformatics* 2011;27(12):1739-40. doi: 10.1093/bioinformatics/btr260 [published Online First: 2011/05/07]
